# Genetic loss of the dopamine transporter significantly impacts behavioral and molecular responses to sub-chronic stress in mice

**DOI:** 10.3389/fnmol.2024.1315366

**Published:** 2024-02-29

**Authors:** Avelina Petri, Abigail Sullivan, Kristen Allen, Benjamin D. Sachs

**Affiliations:** Department of Psychological and Brain Sciences, Villanova University, Villanova, PA, United States

**Keywords:** resilience, stress, dopamine, sex differences, anxiety

## Abstract

Dopaminergic neurotransmission has emerged as a critical determinant of stress susceptibility and resilience. Although the dopamine transporter (DAT) is known to play a key role in maintaining dopamine (DA) homeostasis, its importance for the regulation of stress susceptibility remains largely unknown. Indeed, while numerous studies have examined the neurochemical and behavioral consequences of genetic loss of DAT, very few have compared responses to stress in wild-type and DAT-knockout (KO) animals. The current study compared the responses of male and female WT and DAT-KO mice to a model of sub-chronic stress. Our results reveal that DAT-KO mice are resistant to stress-induced increases in the latency to enter the light chamber of the light-dark emergence test and demonstrate that DAT-KO mice exhibit baseline reductions in forced swim test immobility and grooming time in the splash test of grooming behavior. In addition to these behavioral changes, our results highlight the importance of sex and dopaminergic neurotransmission on stress-induced changes in the expression and phosphorylation of several signal transduction molecules in the nucleus accumbens that have previously been implicated in the regulation of stress susceptibility, including ERK, GSK3β, and ΔFosB. Overall, these results provide further evidence of the importance of dopaminergic neurotransmission in regulating stress susceptibility and suggest that genetic loss of DAT prevents stress-induced increases in anxiety-like behavior.

## Introduction

Stress is known to negatively impact mental health (Kendler et al., 1999; Platt et al., 2016), but only a subset of individuals exposed to stress develop pathological cognitive and behavioral alterations that lead to a diagnosis of mental illness. Understanding the basis for individual differences in stress susceptibility has the potential to help uncover new approaches to promote resilience to and recovery from stress-related psychological disorders. The mesolimbic reward circuit, which consists of dopaminergic projections from the ventral tegmental area (VTA) to the nucleus accumbens (NAc) and amygdala (Nestler and Carlezon, 2006; Salgado and Kaplitt, 2015), has been heavily implicated in stress responses (Dutcher and Creswell, 2018). Preclinical studies have shown that stress-susceptible mice exhibit distinct alterations in gene expression and signal transduction in these brain regions compared to stress-resilient animals following several types of stress, including social defeat stress (SDS) and sub-chronic variable stress (SCVS; Berton et al., 2006; Krishnan et al., 2007, 2008; Hodes et al., 2015; Bagot et al., 2016). Furthermore, manipulating the activity of individual nodes within this circuit can promote resilience to stress (Tye et al., 2013; Francis et al., 2015), highlighting its critical role in stress responses. However, separate lines of evidence have suggested that the nigrostriatal, mesolimbic, and mesocortical dopamine pathways can all influence anxiety-like behavior (Zarrindast and Khakpai, 2015).

One major regulator of DA neurotransmission is the DA transporter (DAT; Gainetdinov et al., 1998). Genetic loss of DAT has been shown to increase extracellular DA levels ~5-fold, to influence synaptic plasticity in the NAc (Yao et al., 2004), and to alter neural activity in several brain regions (Dzirasa et al., 2009a,b; Zhang et al., 2010). Both genetic and pharmacologic inhibition of DAT have been reported to induce behavioral disturbances in rodents, most notably hyperactivity (Giros et al., 1996; Gainetdinov et al., 1999a). DAT-KO mice have also been reported to exhibit increased stereotyped, inflexible behavior (Pogorelov et al., 2005) and significant reductions in immobility time in the forced swim test (FST; Perona et al., 2008), a phenotype that has been suggested to reflect impaired stress coping (Spielewoy et al., 2000). However, we are unaware of studies that have compared DAT-KO to wild-type (WT) mice in established stress paradigms, such as SDS (Golden et al., 2011), chronic mild stress (CMS; Willner, 2005), or SCVS (Hodes et al., 2015). Two studies have examined the effects of acute stress in WT and DAT-KO rats, and both suggest that stress responses are significantly altered by the loss of DAT. Specifically, one study reported that stress exacerbates the hyperactivity phenotype of DAT-KO animals compared to WT rats (Mariano et al., 2020), while the other reported that DAT-KO animals have altered corticosterone levels both at baseline and following acute restraint stress (Illiano et al., 2020). In keeping with a potential role for DAT in stress susceptibility, prior work has reported that cocaine, a non-specific pharmacological inhibitor of DAT, increases vulnerability to SDS (Covington et al., 2011).

Increasing dopaminergic neurotransmission could potentially impact stress responses through multiple cellular and molecular mechanisms. Signaling through D2 receptors has been shown to increase the activation of both ERK (Cai et al., 2000) and GSK3β (Beaulieu et al., 2007) signaling, while signaling through D1 receptors has been reported to increase ΔFosB expression (Krapacher et al., 2022). Similarly, stress has been shown to impact each of these signaling molecules as well (Krishnan et al., 2007; Vialou et al., 2010). Several models of sub-chronic stress have been developed recently and shown to impact neural activity and gene expression in the NAc (Hodes et al., 2015; Brancato et al., 2017; Baugher et al., 2022). However, sub-chronic variable stressors have been shown to impact other structures and circuits as well, including projections from the lateral habenula to the VTA (Zhang et al., 2018), cholinergic neurons in the nucleus basalis of Meynert (Eck et al., 2020), and microglial activation in both the NAc and the hippocampus (Tsyglakova et al., 2021). The 5 day stress (5DS) model used here has been reported to induce significant behavioral alterations in both male and female c57BL6 animals in traditional measures of depression- and/or anxiety-like behavior (Baugher et al., 2022). The current study aimed to determine the impact of global loss of DAT on responses to 5DS. In addition to evaluating behavior, we compared the activity of several signaling pathways known to be impacted by stress and/or DA in the NAc, including the ERK, GSK3, and ΔFosB pathways. Our results demonstrate that DAT-KO mice are partially resilient to stress-induced alterations in light-dark emergence test (LDE) behavior, and they exhibit aberrant behavior in the FST and splash test of grooming behavior (STGB) regardless of stress. Our molecular findings provide novel insight into the complex regulation of several signaling pathways implicated in stress susceptibility. Indeed, while prior studies reported that stress can impact the expression and/or phosphorylation of ΔFosB (Vialou et al., 2010), GSK3β (Krishnan et al., 2007; Wilkinson et al., 2011), and ERK (Krishnan et al., 2007), our current results reveal significant sex and genotype differences in the effects of stress on these important signaling molecules. Together, our findings of aberrant behavioral and molecular adaptations to stress in DAT-KO mice suggest that genetic loss of DAT could protect against the anxiogenic effects of stress.

## Methods

### Animals

Homozygous wildtype (WT) and DAT-KO mice (both males and females) were used in this study. The DAT-KO line was backcrossed onto a c57Bl6 background for 10 generations but has been maintained as a separate breeding line for over a decade. Mice were between 8 and 16 weeks old at the start of behavioral testing. Mice were age-matched across groups, as littermates were used as controls. These animals were generated from heterozygous breeding pairs at Villanova University. Food and water were available *ad libitum* other than during stress exposure and behavioral testing, and the animals were housed in a temperature- and humidity-controlled room on a 12-h light/dark cycle. The mice were housed two-five per cage in standard mouse cages (7$\frac{1}{2}$” W x 11$\frac{1}{2}$” L x 5” H). Males weighed more than females [main effect of sex, *F*_(1, 81)_ = 50.13, *p* < 0.001, partial Eta squared = 0.404], and WT mice weighed more than KO animals [main effect of genotype, *F*_(1, 81)_ = 38.84, *p* < 0.001, partial Eta squared = 0.344], but there were no differences in body weight between stress-exposed and control animals. All studies were performed in accordance with the Guide for the Care and Use of Animals in Research and were covered by protocols that were approved by the IACUC.

### Five day stress

For the stress condition, mice were either subjected to the 5DS paradigm or were handled daily as controls, as described previously (Baugher et al., 2022). Restraint occurred in ventilated 50 ml conical tubes for 1 h daily on days 1 and 4. Tail suspension occurred for 1 h on day 3, and forced swimming occurred in 25°C water for 12 min on days 2 and 5. To control for differences in access to food and water, control animals were denied access to food and water during the daily periods of stress exposure for the 5DS groups.

### Behavior testing

**Figure 1:**
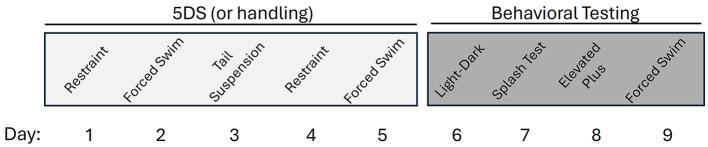
Timeline of stress and behavioral testing.

Starting 1 day after the end of stress, mice were tested on the following schedule: day 6, light-dark emergence test (LDE); day 7, splash test of grooming behavior (STGB); day 8, elevated plus maze (EPM); day 9, forced swim test (FST). The timeline of stress exposure and behavioral testing is shown in Figure 1. Scheduling of tests was intended to ensure that more stressful tests were conducted last to minimize carry-over effects. This schedule is also generally consistent with several prior publications using similar methods (Hodes et al., 2015; Baugher et al., 2022).

### Light-dark emergence test

The light-dark emergence test (LDE) was performed over a 5-min period as described previously (Baugher et al., 2022). Briefly, mice were placed into the dark side of the light-dark box. ANY-maze tracking software was used to measure time, distance, and entries in the light compartment of the box, as well as the latency to enter the light compartment. The light compartment was illuminated at ~400 lux. The LDE is generally considered to be a measure of anxiety-like behavior that is sensitive to the action of anxiolytics (Crawley and Goodwin, 1980).

### Splash test of grooming behavior

The splash test of grooming behavior (STGB) was performed as described previously (Baugher et al., 2022) under red light (in the dark phase). Briefly, mice were transferred into a new, clean cage with standard bedding, where they were sprayed with 10% sucrose. Each mouse's behavior was digitally recorded for 5 min after being sprayed. Observers unaware of the sex, genotype, and stress status of each mouse measured the amount of time that each mouse spent grooming. The STGB is thought to model impairments in self-care that are sometimes associated with depression (Griebel et al., 2002).

### Elevated plus maze

The EPM was performed as described previously using ANY-maze animal tracking software (Baugher et al., 2022) at a lighting level of ~240 lux. The location and activity of the mice was measured for 5 min. Mice that jumped or fell from the open arms of the EPM were excluded from the analysis. The EPM has been validated as a model of anxiety-like behavior in rodents (Pellow et al., 1985; Lister, 1987).

### Forced swim test

The FST was performed as we have described previously (Baugher et al., 2022) at a lighting level of ~240 lux. Briefly, mice were placed in a 4L beaker filled with ~2,500 ml of 25°C water, and their behavior (immobility time, number of immobile episodes, distance swam, and immobility latency) was recorded for 6 min using ANY-maze software. Interpretation of FST data is controversial, as immobility in this test has been suggested to provide insight into despair-like behavior (Porsolt et al., 1977), anxiety-like behavior (Anyan and Amir, 2018), stress coping strategies (Commons et al., 2017), and psychomotor retardation (Unal and Canbeyli, 2019).

### Sample collection

On day 10, the day following the last behavior test, the mice were euthanized by cervical dislocation followed by decapitation. The brains were removed rapidly and placed in an ice-cold 1-mm coronal mouse brain matrix. Sections of brain (1 mm thickness) were cut using razor blades and then placed on an ice-cold metal block. A 1.5 mm biopsy punch was used to collect the NAc from each hemisphere. Punches were collected from ~1.7 mm anterior to bregma to ~0.7 mm anterior to bregma. While centered on the NAc, some of the ventral striatum was also present in these samples. These samples were immediately frozen on dry ice then stored at −80°C.

### Western blotting

Brain samples were lysed with a Tris lysis buffer containing Halt™ Protease and Phosphatase Inhibitors. After boiling in Laemmli buffer, lysates were loaded onto 10% Mini-PROTEAN TGX precast gels and transferred to PVDF membranes. Membranes were washed in TBS-Tween (TBS-T) and blocked with 5% non-fat milk in TBS-T before being treated with one of the following primary antibodies overnight at 4°C: anti-Δ FosB (Cell Signaling Technologies, #14695, 1:500 dilution); anti-phospho-ERK (Cell Signaling Technologies, #4370, 1:300); anti-total-ERK (Cell Signaling Technologies, #9107, 1:500); anti-α-phospho-GSK3-β (Cell Signaling Technologies, #9323, 1:500); anti-total-GSK3 (Cell Signaling Technologies, #9832, 1:300); and anti-GAPDH (Santa Cruz Biotechnology, #sc32233, 1:500). After washing in TBS-T, the appropriate secondary antibody was applied. The secondary antibodies were Rockland goat anti-rabbit DyLight™ 680 and Rockland goat anti-mouse DyLight™ 680 in 5% milk in TBS-T solution. Between primary antibodies the membranes were stripped with 0.1 M glycine (pH 2.5), washed with 1% SDS, then blocked in 5% non-fat milk in TBS-T. Densitometry was performed using ImageJ and values were normalized to GAPDH. Male and female samples were run on different gels, and the WT male control and the WT female control groups were separately normalized to a value of 1.0 arbitrary units.

### Statistical analysis

Data were analyzed by three-way ANOVAs with genotype (WT or KO), stress (control or 5DS), and sex (male or female) as factors using JMP Pro 16 software. Tukey's *post-hoc* tests were used to determine individual group differences. However, for the Western blot analyses, main effects of sex and *post-hoc* comparisons between the sexes were not considered, as the relative protein levels were not directly compared across sexes. Effect size testing was performed using SPSS software (Version 29.0). *P*-values < 0.05 were considered statistically significant.

## Results

### Light-dark emergence test

**Figure 2:**
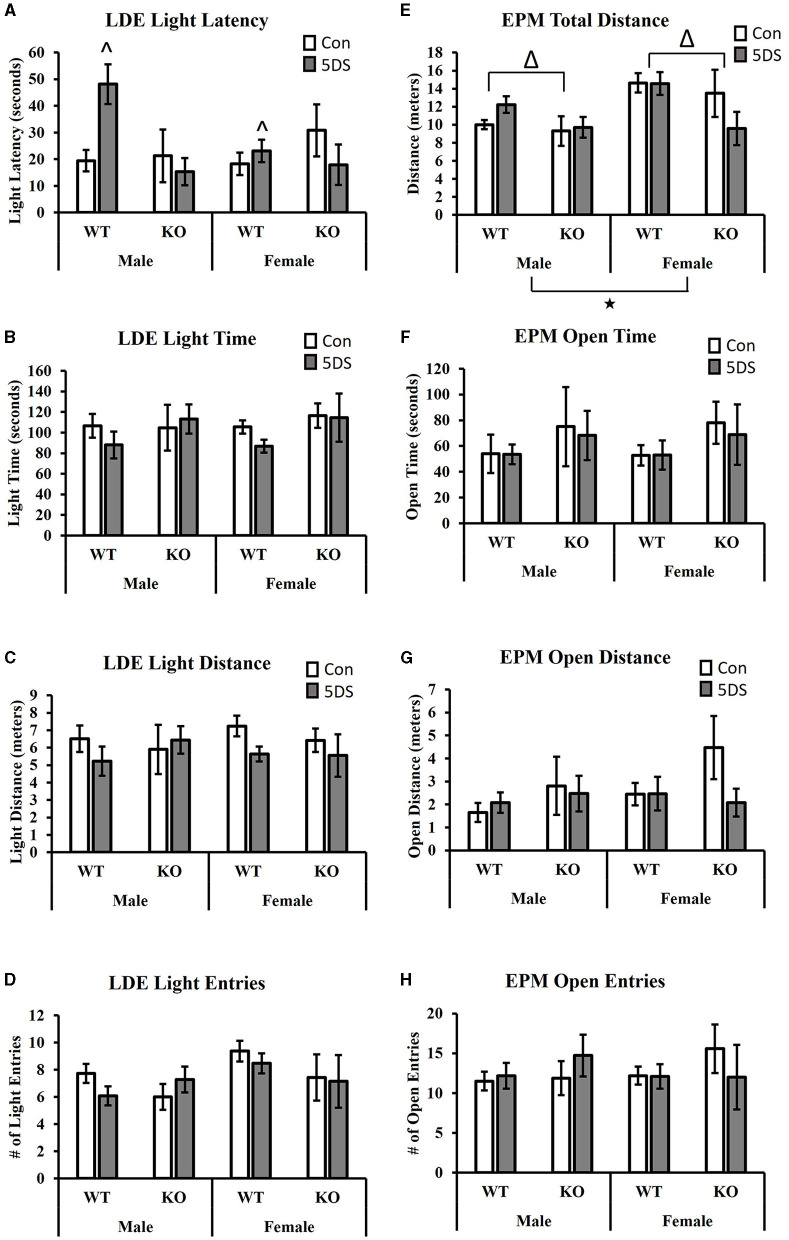
Effects of 5DS on light-dark emergence (LDE) and elevated plus maze (EPM) behavior in WT and DAT-KO mice. Latency to enter the light chamber in the LDE **(A)**. Time spent in the light chamber in the LDE **(B)**. Distance traveled in the light chamber **(C)**. Number of entries into the light chamber **(D)**. Total distance traveled in the EPM **(E)**. Time spent in the open arm in the EPM **(F)**. The distance traveled in the open arm **(G)**. The number of entries into the open arm **(H)**. Significant two-way interactions between genotype and sex and genotype and stress were observed in **(A)**. “^∧^” denotes that the 5DS-exposed WT mice have a significantly higher latency than control WT mice (sexes combined) by Tukey's *post-hoc* test. “Δ” indicates an overall main effect of genotype by three-way ANOVA. “⋆” indicates a significant main effect of sex by three-way ANOVA. For the LDE, *N* = 11 for male-WT-control, 12 for male-WT-5DS, seven for male-KO-control, seven for male-KO-5DS, 16 for female-WT-control, 15 for female-WT-5DS, seven for female-KO-control, and seven for female-KO-5DS. For the EPM, *N* = 10 for male-WT-control, 12 for male-WT-5DS, seven for male-KO-control, seven for male-KO-5DS, 16 for female-WT-control, 13 for female-WT-5DS, seven for female-KO-control, and six for female-KO-5DS. Data are shown as the mean +/− the SEM.

In the LDE, a significant genotype by stress interaction was observed for the latency to enter the light chamber [*F*_(1, 80)_ = 8.13, *p* = 0.006, partial Eta squared = 0.099, Figure 2A]. Tukey's *post-hoc* analysis revealed that 5DS significantly increased light latency in WT animals (partial Eta squared = 0.056), but not in KO mice (partial Eta squared = 0.00). A significant sex by genotype interaction was also observed [*F*_(1, 80)_ = 4.35, *p* = 0.04, partial Eta squared = 0.056, Figure 2A], in which male DAT-KOs exhibited a ~54% shorter latency to enter the light than WT males (partial Eta squared = 0.068), but DAT-KO females had a latency that was ~18% longer than WT females (partial Eta squared = 0.005). No significant effects of stress or genotype were observed for the time spent in the light chamber (Figure 2B), the distance in the light chamber (Figure 2C), or the number of entries into the light chamber (Figure 2D).

### Elevated plus maze

When analyzing total distance traveled in the EPM, significant main effects of both sex [*F*_(1, 77)_ = 7.64, *p* = 0.007, partial Eta squared = 0.098] and genotype were observed [*F*_(1, 77)_ = 5.49, *p* = 0.022, partial Eta squared = 0.073 Figure 2E], in which female and WT mice traveled a greater distance than male and DAT-KO mice, respectively. However, no significant effects of stress or genotype were observed for time spent in the open arms (Figure 2F), distance in the open arms (Figure 2G), or the number of open arm entries (Figure 2H).

### Splash test of grooming behavior

**Figure 3:**
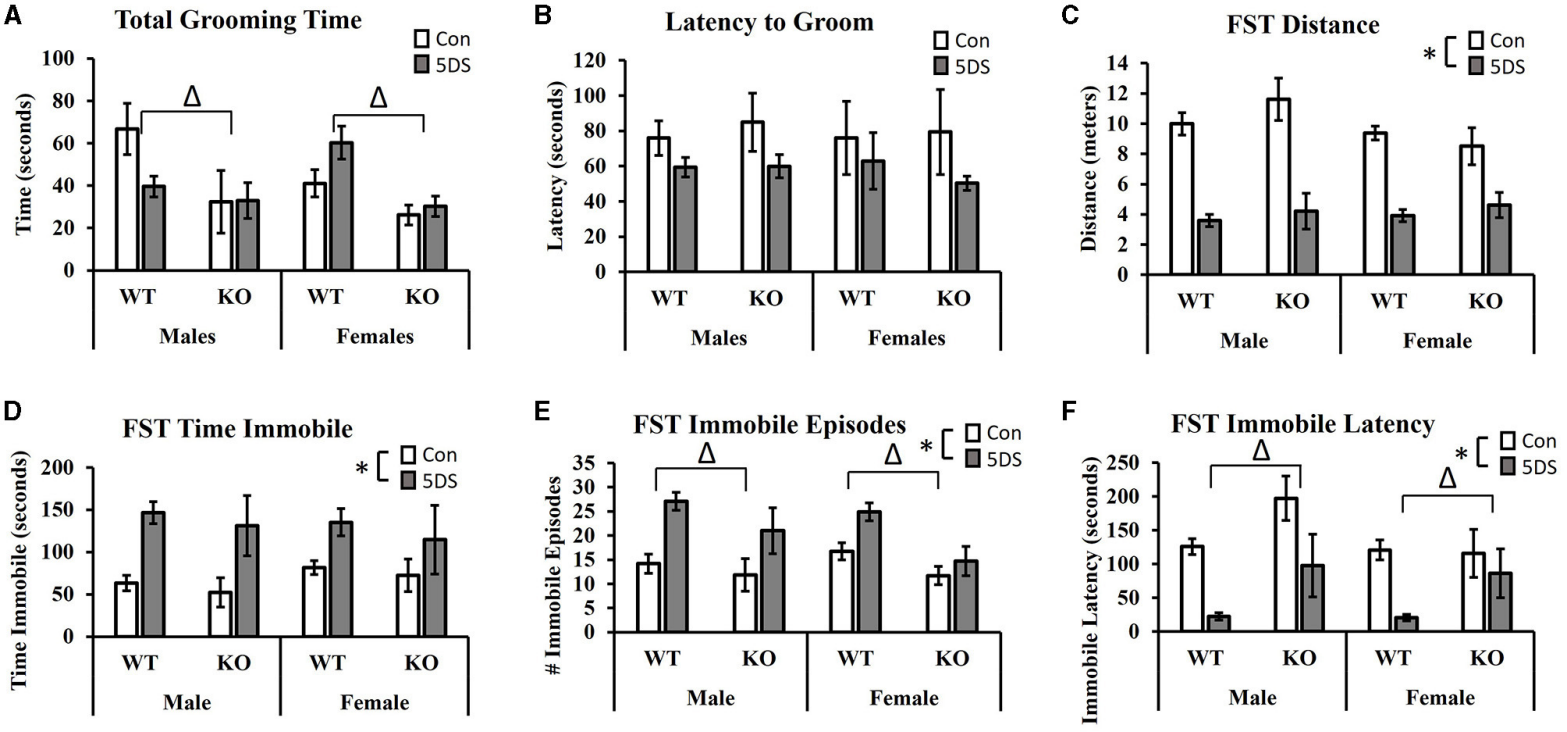
Effects of 5DS on the splash test of grooming behavior (STGB) and on forced swim test (FST) behavior in WT and DAT-KO mice. Total grooming time in the STGB **(A)**. Latency to groom in the STGB **(B)**. Total distance traveled in the FST **(C)**. Time spent immobile in the FST **(D)**. Number of immobile episodes in the FST **(E)**. Latency to first immobility in the FST **(F)**. A significant two-way interaction between stress and sex was observed in C. “Δ” indicates an overall main effect of genotype by three-way ANOVA. “*” indicates a main effect of 5DS by three-way ANOVA. *N* = 11 for male-WT-control, 12 for male-WT-5DS, seven for male-KO-control, seven for male-KO-5DS, 16 for female-WT-control, 15 for female-WT-5DS, seven for female-KO-control, and seven for female-KO-5DS. Data are shown as the mean +/- the SEM.

In the STGB, a significant main effect of genotype was observed on total grooming time [*F*_(1, 80)_ = 11.32, *p* = 0.001, partial Eta squared = 0.134, Figure 3A] in which DAT-KO animals showed a reduction in total grooming time compared to WT mice. There was also a trend toward a stress by sex interaction [*F*_(1, 80)_ = 3.80, *p* = 0.055, partial Eta squared = 0.049, Figure 3A] in which 5DS slightly reduced grooming time in male mice while slightly increasing it in females. Although these trends toward sex-specific effects of 5DS appeared more pronounced in WT than DAT-KO animals, the three-way stress by sex by genotype failed to reach statistical significance [*F*_(1, 80)_ = 2.80, *p* = 0.089, Figure 3A]. When latency to begin grooming was evaluated, there was a trend toward a reduced latency to groom in 5DS-treated animals compared to controls [*F*_(1, 80)_ = 2.84, *p* = 0.096, Figure 3B], but no other significant effects or trends were observed.

### Forced swim test

In the FST, 5DS significantly decreased the total distance traveled in both WT and DAT-KO mice [main effect of stress, *F*_(1, 80)_ = 112.52, *p* < 0.0001, partial Eta squared = 0.603 Figure 3C]. A significant stress by sex interaction was also observed [*F*_(1, 80)_ = 4.15, *p* = 0.0451], in which males were more sensitive to this effect than females (partial Eta squared = 0.523 in males vs. partial Eta squared = 0.374 in females). Significant main effects of 5DS were also observed for time spent immobile [*F*_(1, 80)_ = 22.33, *p* < 0.0001, partial Eta squared = 0.232, Figure 3D], the number of immobile episodes [*F*_(1, 80)_ = 21.83, *p* < 0.0001, partial Eta squared = 0.228, Figure 3E], and latency to first immobile episode [*F*_(1, 80)_ = 29.41, *p* < 0.0001, partial Eta squared = 0.284, Figure 3F]. Although no genotype by stress interactions were observed on any of these dependent variables, significant main effects of genotype were detected for the number of immobile episodes [*F*_(1, 80)_ = 11.07, *p* = 0.001, partial Eta squared = 0.130, Figure 3E] and immobile latency [*F*_(1, 80)_ = 11.39, *p* = 0.001, partial Eta squared = 0.133, Figure 3F], in which KO mice exhibited fewer immobile episodes and a greater latency to first immobility.

### Signal transduction

**Figure 4:**
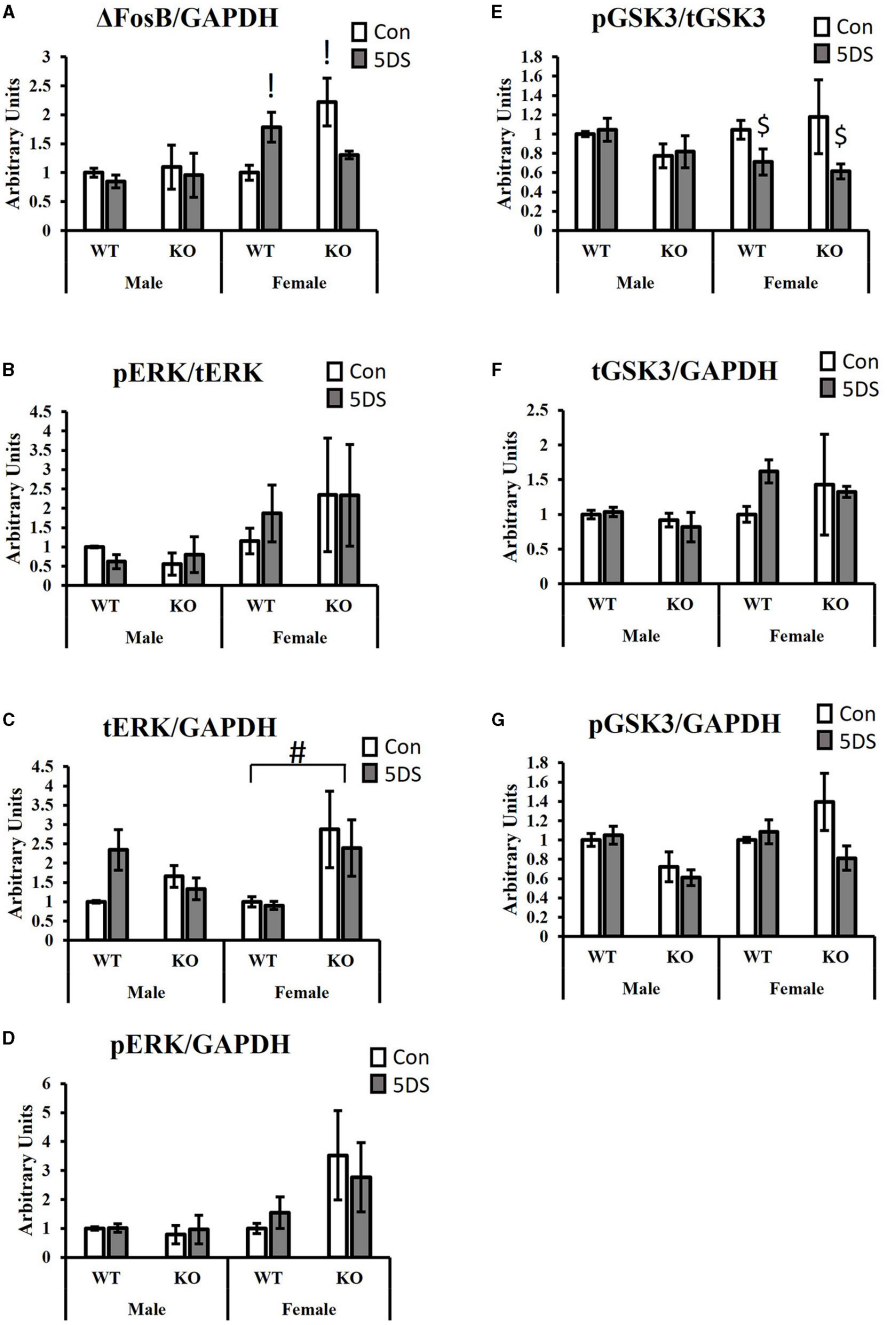
Signaling effects of 5DS in the NAc of WT and DAT-KO mice. ΔFosB expression relative to GAPDH **(A)**. Phospho-ERK relative to total ERK **(B)**. Total ERK relative to GAPDH **(C)**. Phospho-ERK relative to GAPDH **(D)**. Phospho-GSK3β reactive to total GSK3β **(E)**. Total GSK3β relative to GAPDH **(F)**. Phospho-GSK3β relative to GAPDH **(G)**. A significant three-way interaction between genotype, stress, and sex was observed in **(A)**. Significant two-way interactions between genotype and stress were observed for **(A, G**). Significant two-way interactions between genotype and sex were observed for **(C, G)**, and a significant two-way interaction between stress and sex was observed for **(E)**. “G x 5DS” indicates a significant two-way interaction between genotype **(G)** and stress (5DS). “G x” indicates a significant two-way interaction between genotype **(G)** and sex. “5DS x” indicates a significant two-way interaction between stress (5DS) and sex. Both columns marked with “!” are significantly different from WT control female by Tukey's *post-hoc* test. “#” indicates the significant genotype difference in females, but not males determined by Tukey's *post-hoc* test. “$” denotes that 5DS-exposed females are significantly different from control females (with genotypes combined) by Tukey's *post-hoc* test. *N* = 5 for male-WT-control, six for male-WT-5DS, three for male-KO-control, three for male-KO-5DS, five for female-WT-control, five for female-WT-5DS, four for female-KO-control, and four for female-KO-5DS. Data are shown as the mean +/- the SEM.

For ΔFosB levels in the NAc, a significant three-way (genotype by stress by sex) interaction was observed [*F*_(1, 34)_ = 6.87, *p* = 0.014, partial Eta squared = 0.203, Figure 4A], as was a significant genotype by stress interaction [*F*_(1, 34)_ = 6.70, *p* = 0.015, partial Eta squared = 0.199, Figure 4A]. For the three-way interaction, stress tended to increase ΔFosB levels in WT females (partial Eta squared = 0.207) and to decrease it in DAT-KO females (partial Eta squared = 0.219), but minimal effects were observed in both genotypes of males (partial Eta squared in WT males = 0.011 and partial Eta squared = 0.005 in KO males). Tukey's *post-hoc* test revealed that DAT-KO control females had significantly more ΔFosB in the NAc than WT control females (*p* = 0.0123, partial Eta squared = 0.357) and that WT stressed females had significantly more ΔFosB than WT control females (*p* = 0.0462, partial Eta squared = 0.207).

When ERK phosphorylation was evaluated (compared to total ERK), no significant effects were observed (Figure 4B). However, total ERK levels normalized to GAPDH differed between groups. Specifically, DAT-KO mice exhibited a significant increase in total ERK in the NAc compared to WT animals [significant main effect of genotype, *F*_(1, 34)_ = 4.52, *p* = 0.043, partial Eta squared = 0.143, Figure 4C], but a significant genotype by sex interaction was also observed [*F*_(1, 34)_ = 6.88, *p* = 0.014, partial Eta squared = 0.203, Figure 4C] in which this genotype difference was only observed in females (*p* = 0.009, partial Eta squared = 0.31), not in males (*p* = 0.986, partial Eta squared = 0.004). When pERK levels were normalized to GAPDH, there was a trend toward a genotype by stress interaction in which 5DS slightly reduced ERK phosphorylation in DAT-KO mice but slightly increased it in WT animals (*p* = 0.053, partial Eta squared = 0.132, Figure 4D).

Evaluating the levels of phosphorylated GSK3β (pGSK3β) normalized to total GSK3, a significant stress by sex interaction was observed [*F*_(1, 34)_ = 5.03, *p* = 0.034, partial Eta squared = 0.162, Figure 4E] in which stress significantly reduced GSK3β phosphorylation in females (*p* = 0.033, partial Eta squared = 0.248), but not males (*p* = 0.99). No significant group differences in tGSK3β levels compared to GAPDH were observed between groups (Figure 4F). When pGSK3β levels were normalized to GAPDH, significant interactions were observed between genotype and sex [*F*_(1, 34)_ = 4.65, *p* = 0.04, partial Eta squared = 0.148] and between genotype and stress [*F*_(1, 34)_ = 4.58, *p* = 0.042, partial Eta squared = 0.146, Figure 4G]. Tukey's *post-hoc* test revealed no significant group differences for the genotype by stress interaction, but stress-exposed WT mice tended to have more pGSK3β than stress-exposed DAT-KO animals (*p* = 0.06, partial Eta squared = 0.204) whereas no such trend was observed in control animals (*p* = 0.97, partial Eta squared = 0.007). For the genotype by sex interaction, male DAT-KOs tended to have less pGSK3β than male WTs, but this trend was not apparent in females, and none of the within-sex group differences were statistically significant in *post-hoc* testing.

## Discussion

The results we observed here in WT animals from the DAT-KO line (which is on a c57BL6 background), were generally consistent with our previously reported findings in c57BL6 animals (Baugher et al., 2022), although the effects of 5DS in the current work appear somewhat less pronounced than those in prior studies. The current data show that 5DS increases immobility in the FST (with a greater effect in males than females) and the latency to enter the light chamber in the LDE similar to prior work (Baugher et al., 2022). The current results also reveal a trend toward sex-specific effects of 5DS on grooming behavior in the STGB in which 5DS tended to reduce grooming time in males but tended to increase grooming time in females. Stress-induced reductions in grooming are thought to reflect impaired self-care, which could result from a lack of motivation to exert effort to remain well-groomed. If the FST data in which males exhibit a greater reduction of mobility than females is interpreted as reflecting less motivation to exert effort to escape the aversive swim stress, the results from these two tests could be argued to reflect similar phenotypes. However, we acknowledge that there are alternative interpretations of these behavioral effects, particularly for the FST. Regardless, both of these effects were similar to those reported previously in c57BL6 mice (Baugher et al., 2022), although the sex by stress interaction for the STGB did not reach significance here.

Regarding the role of DAT, the current findings suggest that genetic loss of DAT prevents a subset of behavioral consequences of 5DS, most notably the 5DS-induced increase in latency to enter the light chamber in the LDE. Several additional trends toward blunted responses in DAT-KO mice were observed but did not reach statistical significance. For example, for the latency until the first immobile episode in the FST, 5DS reduced immobility latency by more than 80% in both male and female WT mice, but immobility latency was only reduced by ~25 and 50% in female and male DAT-KOs, respectively. Similarly, the effects of 5DS on grooming time in the STGB were minimal in DAT-KO mice, whereas 5DS tended to increase grooming time in WT females and decrease grooming time in WT males (three-way interaction *p*-value = 0.0986). Importantly, DAT-KO mice exhibited an overall reduction in grooming time compared to WT mice in the STGB and displayed less immobility than WT mice in the FST, as had been reported previously (Spielewoy et al., 2000), so these trends toward reduced stress responses in these tests could be largely due to floor effects. Nonetheless, our results suggest that genetic loss of DAT renders mice less sensitive to stress-induced behavioral alterations, most notably in the LDE. Prior work suggests that c57BL6 mice exhibit reduced baseline anxiety compared to other strains, such as C3H and Balb/c (Kopp et al., 1999). It would be interesting to see the effects of genetic loss of DAT in other strains with higher baseline anxiety levels, as other strains may be better suited to observe reduced anxiety. It should also be noted that DAT-KO mice are significantly smaller than their WT counterparts. The extent to which the size or weight of animals impacts sensitivity to 5DS is not known, but it is possible that body weight influences the severity of several stressors, including tail suspension. Similarly, total body size and surface area could influence sensitivity to the effects of forced swimming (due to heat loss) and restraint (due to variation in how tightly animals are restrained). The results in the FST and LDE are generally consistent with prior work showing that knocking down the expression of DAT in the NAc or knocking out DAT induces antidepressant- and anxiolytic-like effects (Spielewoy et al., 2000; Bahi and Dreyer, 2019). In contrast, stress-induced reductions in grooming are generally thought to reflect a pro-depression-like effect (Griebel et al., 2002; Santarelli et al., 2003; Hodes et al., 2015). Applying this interpretation to the reduced grooming phenotype of DAT-KO mice would suggest that these mice exhibit increased depression-like behavior, but we think it is more likely that the reduction in grooming is a secondary consequence of their hyperactivity, not the emergence of an apathy-like state relevant to depression-like behavior.

Given that 5DS-exposed animals had a history of exposure to forced swimming, their increased immobility in the FST is likely driven at least in part by an experience-driven change in stress coping, not the emergence of a despair-like phenotype (Baugher et al., 2022). We acknowledge that there are many possible interpretations of FST immobility, including despair (Porsolt et al., 1977) or psychomotor retardation (Unal and Canbeyli, 2019), the lack of anxiety (Anyan and Amir, 2018), and passive stress coping (Commons et al., 2017). Regardless of the interpretation, loss of DAT did not significantly impact the effects of stress on FST behavior, although DAT-KOs did exhibit fewer immobile episodes and a longer latency to first immobility overall. However, consistent with our previous work (Baugher et al., 2022) and work from other groups (Colom-Lapetina et al., 2017), females exhibited a smaller increase in immobility following prior swimming exposure than males. Whether hormonal differences between the sexes contribute to this differential susceptibility to stress-induced changes in forced swimming immobility and whether this effect is estrus-cycle-dependent would require additional study.

The lack of a 5DS-induced increase in latency to enter the light in the LDE in DAT-KO animals could reflect the lack of a stress-induced maladaptive increase in anxiety, or the lack of an adaptive behavioral adaptation following adverse experience, or perhaps even the emergence of a stress-induced increase in impulsive, risk-taking behavior. DAT-KO animals have previously been suggested to demonstrate anti-anxiety-like phenotypes (Spielewoy et al., 2000; Bahi and Dreyer, 2019) along with phenotypes related to behavioral inflexibility (Pogorelov et al., 2005), and conditions characterized by increased impulsivity, such as manic episodes (van Enkhuizen et al., 2014, 2015) and attention deficit hyperactivity disorder (Gainetdinov et al., 1999a,b; Regan et al., 2022). Currently, it remains unclear whether the observed phenotype in DAT-KOs (or lack thereof) reflects a protective anxiolytic effect or a maladaptive manifestation of behavioral inflexibility or the emergence of excessive impulsivity.

Prior research suggests that mice that are resilient to SDS exhibit increased expression of ΔFosB in the NAc compared to susceptible animals (Vialou et al., 2010). The current finding of elevated levels of ΔFosB in the NAc of DAT-KO mice, which are partially resistant to 5DS, is consistent with this. However, the increased ΔFosB was only observed in DAT-KO females, whereas resilience was observed in both sexes. Thus, it is likely that resilience of DAT-KO mice is not entirely mediated by increased ΔFosB (at least in males). One prior study in male DAT-KO rats reported no significant increase in ΔFosB expression in the NAc core (Sanna et al., 2020). Rather, significantly elevated levels of ΔFosB were observed in the VTA of male DAT-KO rats along with significant reductions in ΔFosB levels in the medial prefrontal cortex and shell of the NAc (Sanna et al., 2020). Future research would be required to determine the importance of the region-specific alterations in ΔFosB levels in DAT-KO animals and the mechanisms underlying the observed sex differences in ΔFosB regulation by DA.

Like ΔFosB, GSK3β has been implicated in stress responses. Although most work suggests that GSK3β inhibition promotes antidepressant-like effects, several conflicting reports have also been published. Consistent with the idea that increased GSK3β activity is associated with “depression-like” behavior, mice expressing a mutant form of GSK3β that is insensitive to inhibitory phosphorylation have been reported to exhibit increased immobility in the FST, reduced time spent in the open arms of the EPM, and increased susceptibility to stress (Polter et al., 2010). Separate work has shown that reducing GSK3β expression in the hippocampus via shRNA-mediated knockdown induces antidepressant-like effects in mice exposed to stress (Omata et al., 2011). Similarly, mice that are susceptible to SDS have been reported to express less phosphorylation of GSK3β in the NAc (i.e., more GSK3 activity) compared to control animals (Wilkinson et al., 2011). However, another study reported essentially the opposite: that SDS-susceptible mice exhibit a significant *increase* in GSK3β phosphorylation in the NAc compared to resilient mice and non-stressed controls (Krishnan et al., 2007). The reasons for these conflicting results are not clear. Our results reveal a stress by sex interaction in which stress reduced the pGSK3/tGSK3 ratio in females, but not males. Given that we did not observe any significant overall sex differences in susceptibility to 5DS in the assays tested here, it is possible that the distinct molecular alterations observed in males and females reflect an example of sexual convergence, in which similar phenotypes emerge in response to unique molecular pathology in males and females. One limitation of the current study is that we did not evaluate potential estrus cycle effects on the sex differences observed here, so it is unclear whether the sex differences in stress-induced alterations in GSK3 signaling occur regardless of estrus cycle stage, but the estrus cycle has been reported to regulate brain GSK3 signaling (Barrera-Ocampo et al., 2012).

Finally, mice that are susceptible to SDS have been reported to exhibit significantly elevated levels of pERK in the NAc ~48 h after the final stress exposure (Krishnan et al., 2007). The current results do not reveal any significant stress-induced changes in ERK phosphorylation in the NAc, but it is important to note that there are significant procedural differences between these studies, which use different stressors (5DS vs. SDS) and compared ERK phosphorylation at different time points (5 vs. 2 days) following stress. Our results did reveal that DAT-KO females express more total ERK than WT females, an effect not observed in males. One previous study reported increased pERK in the striatum of DAT-KO animals (Beaulieu et al., 2006), but the current finding reveals an increase in tERK (and only a trend toward increased pERK), whereas the prior study reported an increase in pERK in the absence of any significant alterations in tERK (Beaulieu et al., 2006). Nonetheless, an increase in ERK activity in response to DAT inhibition appears consistent across these studies. One limitation of the present molecular results is that by running separate Western blots for males and females, we are unable to determine whether there are overall sex differences in the levels of any of the signaling molecules examined. Nonetheless, our analyses do reveal differential effects of genotype and 5DS in males and females. Future work should continue to explore additional potential sex differences in signal transduction at baseline and in response to genetic and environmental manipulations.

Overall, our results provide further support for the important role of dopaminergic neurotransmission in influencing stress responses by demonstrating that DAT-KO mice are partially resistant to stress-induced increases in anxiety-like behavior. The absence of stress-induced behavioral alterations is typically interpreted as beneficial (i.e., pro-resilience), and our current interpretation of the present results is that DAT-KO animals display partial stress resilience. However, we acknowledge that behavioral inflexibility or an increase in impulsivity could also explain the phenotype observed here. It is also important to note that at least some behavioral adaptations to stress are likely to be adaptive. If the behavioral alterations induced by sub-chronic stress in WT animals constitute an adaptive response to repeated adverse events (i.e., adaptive anxiety vs. pathological anxiety), then it is possible that DAT-KO animals are not appropriately adapting to environmental conditions. Future research could provide additional insight into whether the behavioral alterations of DAT-KO mice are beneficial vs. deleterious by performing more comprehensive behavioral testing and by evaluating the ability of specific drug classes (e.g., antidepressants or mood stabilizers) to reverse stress-induced phenotypes. Regardless, our results provide new insight into the importance of DAT in regulating stress responses and highlight the need for additional research to better understand the ways in which manipulating dopaminergic neurotransmission could reduce the risk of stress-related disorders.

## Data availability statement

The raw data supporting the conclusions of this article will be made available by the authors, without undue reservation.

## Ethics statement

The animal study was approved by Villanova University's Institutional Animal Care and Use Committee. The study was conducted in accordance with the local legislation and institutional requirements and the Guide for the Care and Use of Laboratory Animals.

## Author contributions

AP: Formal analysis, Investigation, Methodology, Visualization, Writing – original draft, Writing – review & editing. AS: Formal analysis, Investigation, Methodology, Visualization, Writing – original draft, Writing – review & editing. KA: Investigation, Methodology, Writing – review & editing. BS: Conceptualization, Formal analysis, Investigation, Methodology, Project administration, Resources, Supervision, Visualization, Writing – original draft, Writing – review & editing.
